# Treatment of insomnia in myasthenia gravis—A prospective study on non-benzodiazepine hypnotics in the treatment of myasthenia gravis patients with insomnia

**DOI:** 10.3389/fneur.2023.1266862

**Published:** 2023-09-22

**Authors:** Jingwen Yan, Minhui Yang, Michael Ke Li, Yangyu Huang, Ying Tan, Jiayu Shi, Qianqian Fan, Zhu Zhu, Yuzhou Guan, Liying Cui

**Affiliations:** ^1^Department of Neurology, Peking Union Medical College Hospital, Chinese Academy of Medical Sciences, Beijing, China; ^2^Department of Neurology, Central South University Xiangya School of Medicine Affiliated Haikou Hospital, Haikou, China; ^3^Department of Pharmacy, Peking Union Medical College Hospital, Chinese Academy of Medical Sciences, Beijing, China

**Keywords:** myasthenia gravis, insomnia, non-benzodiazepine hypnotics, zopiclone, zolpidem

## Abstract

**Objectives:**

This study aimed to evaluate the efficacy and safety of non-benzodiazepine hypnotics in the treatment of myasthenia gravis (MG) patients with insomnia.

**Methods:**

This is a prospective longitudinal study. Outpatients who met the criteria for stable MG and insomnia diagnosis according to the International Classification of Sleep Disorders (third edition) were included in the study. They took a regular dose of non-benzodiazepine hypnotics (zolpidem 10 mg per night or zopiclone 7.5 mg per night) based on their own preferences. Patients received psychotherapy (including sleep health education) and were followed up for 4–5 weeks. Cases with lung diseases, respiratory disorders, or inappropriate use of hypnotic medications were excluded. The primary outcome is the difference in total Pittsburgh Sleep Quality Index (PSQI) score between baseline and the end of follow-up period. Secondary outcomes include the difference in Myasthenia Gravis Activities of Daily Living (MG-ADL) score, 7-item Generalized Anxiety Disorder Questionnaire (GAD-7), and the Patient Health Questionnaire-9 (PHQ-9) between baseline and the end of follow-up period and the safety of medication.

**Results:**

A total of 75 MG patients with insomnia were included in this study. After 4–5 weeks of treatment, the total PSQI score and MG-ADL score were lower than baseline (*p* < 0.01). No patients had an increased MG-ADL score. The incidence rate of adverse events was 16.0% (12 cases), including dizziness (6 cases, 8.0%), drowsiness (3 cases, 4.0%), fatigue (2 cases, 2.7%), and nausea (1 case, 1.3%), all of which were mild. No patients had new onset breathing disorders.

**Conclusion:**

Non-benzodiazepine hypnotics are safe and effective for stable MG patients who need insomnia treatment.

## Introduction

Myasthenia gravis (MG) is an antibody-mediated, cellular immune-dependent, complement-involved acquired autoimmune disease. The neuromuscular junction damage results in skeletal muscle weakness (1). Many MG patients also suffer from mental health issues such as depression, anxiety, and insomnia. This could lead to exacerbation of MG or prolonged treatment. The factors of MG patients co-morbid with insomnia include age, respiratory distress, presence of thymoma, 1-month post-operation, and high glucocorticoid dosage (2). Long-term insomnia can aggravate MG and even lead to myasthenia gravis crisis (3). However, some insomnia medications have the potential risk to aggravate MG. There is an urgent need to find safe medications for MG patients with insomnia.

It is generally recommended that patients with MG should not take benzodiazepine and non-benzodiazepine hypnotics (4). Molenaar and Van Kempen (5) used the rat model of MG to study the effect of diazepam on muscle weakness. They found that diazepam does not aggravate muscle weakness caused by neuromuscular blockade. There was a case report (4) that patients with stable MG were treated with benzodiazepines for 11 years without aggravation. In clinical settings, the application of non-benzodiazepine hypnotics in MG patients with insomnia under stable disease control could improve the quality of life while not causing muscle weakness or respiratory disturbances. It has also been reported that (6) the application of the atypical tetracyclic antidepressant mianserin hydrochloride was effective in MG patients with respiratory failure co-morbid with acute insomnia and anxiety.

The development of sedative-hypnotics has gone through barbiturates (the first generation), benzodiazepines (the second generation), and non-benzodiazepine sedative-hypnotics (the third generation). γ-aminobutyric acid (GABA) is an inhibitory neurotransmitter. Non-benzodiazepines (non-BZDs) selectively agonize the α-subunit on the GABAa receptor. Because of its stronger selectivity for the benzodiazepine receptor 1 (BZ1) subunit than BZ2, it reduces the adverse effects of sedative-hypnotic drugs (7). Commonly used non-benzodiazepine drugs include zopiclone, zolpidem, and zaleplon. Zaleplon has a short half-life of 1 h and should only be used in patients who have difficulty falling asleep. Zolpidem has a half-life of 1.5–3.5 h. Zopiclone has a half-life of 3.5–5 h. Compared with benzodiazepines, non-benzodiazepines have the following advantages: low risk of drug dependence and withdrawal symptoms, small muscle weakness effects, short half-lives (≤6 h), small residual effects on the following day, and no daytime sleepiness.

At present, there is only limited research related to sedative-hypnotics for the treatment of MG combined with insomnia. Most of them are case reports, lacking large sample studies. Therefore, we conducted a prospective cohort study of patients with MG combined with insomnia. This study aimed to evaluate the safety and efficacy of sedative-hypnotic medication in MG patients with insomnia.

## Methods

### Patient selection and methods

We consecutively included patients aged 18–85 years old from January 2018 to January 2022 in Peking Union Medical College Hospital, who were diagnosed with MG and insomnia. MG diagnosis was made based on Chinese Guidelines for the Diagnosis and Treatment of Myasthenia Gravis (2015) (1). MG symptoms are stable (MG-ADL ≤ 1). The diagnosis of insomnia is in accordance with the International Classification of Sleep Disorders, third edition (8) (ICSD-3) 2014. The diagnosis of insomnia includes (1) the presence of difficulty falling asleep, difficulty maintaining sleep, or early awakening; (2) daytime fatigue, drowsiness, and impaired social functioning; and (3) the presence of these symptoms at least three times a week for at least 3 months. If the duration of the disease is <3 months, it is called short-term insomnia disorder.

The exclusion criteria include patients who have used other sedative-hypnotics such as benzodiazepines or non-benzodiazepines or Chinese medicines within 1–3 weeks (depending on the half-life of the hypnotic medication) before enrollment. Patients were also excluded if they use other drugs for insomnia (including Chinese medicines with hypnotic effects) during the study period, central nervous system active drugs (antidepressants, anxiolytics, lithium salts, and other prohibited combinations), other psychiatric drugs; have known allergy to zolpidem/zopiclone and sedative-hypnotics; have psychiatric disorders; have vital organ dysfunctions such as the liver, kidney, brain, and heart; have co-infections; have malignancies; have a history of alcohol or drug abuse, drug dependence, and drug addiction; and have any other disease or psychiatric condition that may affect the study results.

### Study design

All patients were treated with psychotherapy (including sleep education). Seventy-five patients were administered non-benzodiazepine (zolpidem 10 mg or zopiclone 7.5 mg once per night) with a fixed dose based on their own preferences. The difference in PSQI between baseline and 4th week of treatment was used as the primary outcome. The difference in MG-ADL, GAD-7, and PHQ-9 scores was used as the secondary outcome. Researchers assessed the outcome indicators at the baseline and the end of the 4th week of treatment, monitored adverse effects, and performed control analysis before and after treatment and group comparison analysis between the groups to evaluate the safety and efficacy. The causal relationship between medication and adverse events was evaluated and determined by applying Noah's assessment scale. The study was approved by the Ethics Committee of Peking Union Medical College Hospital (Ethics Approval Number: I-22PJ189), and informed consent was obtained.

Medication administration is as follows. Zolpidem is produced by Sanofi (Hangzhou) Pharmaceutical Co., Ltd. under the trade name Sinox, with a specification of 10 mg/tablet, 7 tablets per box. Zopiclone tablets were manufactured by Qilu Pharmaceutical Co., Ltd, trade name Sanchen, with a specification of 7.5 mg/tablet, 12 tablets per box. Zolpidem/zopiclone treatment in the study was a fixed dose, i.e., zolpidem 10 mg/zopiclone 7.5 mg orally per night, and no dose adjustment was generally allowed. Temporary adjustment of one study drug (discontinuation of one study drug) was allowed if the subject had an adverse event, but if the adverse event still occurred when the fixed dose of the study drug was taken again, the dose could not be adjusted again; otherwise, the subject had to withdraw from the study. There were three cases of patients over 65 years of age with a starting dose of zolpidem 5 mg/zopiclone 3.75 mg, which was increased to 10/7.5 mg the next day.

Information was collected including patient's gender, age, disease course of MG at the first visit for insomnia, MGFA level, co-morbidities (diabetes, hypertension, and cardiovascular disease), history of thymoma/hyperplasia, history of thymic surgery, total PSQI score, scores for PSQI components 2, 5, and 7, MG-ADL score, GAD-7 score, PHQ-9 score, and total and average daily dosage of prednisone and pyridostigmine bromide. Information was collected at the end of the follow-up period: total PSQI score, scores of PSQI components 2, 5, and 7, MG-ADL score, GAD-7 score, PHQ-9 score, and adverse events.

### Efficacy and safety indicators

1. Difference in PSQI (PSQI at baseline subtracts PSQI at the end of follow up period). The PSQI has a score of 0–3 for each dimension and a total score of 21 (9, 10). A PSQI score of 6–10 is considered as mild sleep disorder, 10–15 as moderate sleep disorder, and 15 or more as severe sleep disorder (11). Component 2 evaluates the time to fall asleep, component 5 evaluates sleep disturbance, and component 7 evaluates daytime function.
2. MG Quality of Daily Living Scale (12, 13) (Myasthenia Gravis Activities of Daily Living, MG-ADL). As a tool to assess the improvement of the subject's ability to live, changes in MG-ADL scores from baseline can reflect changes in the subject's ability to live (safety evaluation).
3. Generalized Anxiety Disorder Questionnaire [7-item Generalized Anxiety Disorder Questionnaire, GAD-7 (14–16)].
4. The 9-item Patient Health Questionnaire-9 (PHQ-9) (17). A 9-item questionnaire that includes the DSM-4 diagnostic criteria for depressive disorders can be used to assess both depression severity and potential diagnostic validity.
5. Self-made adverse events scale. Whether new symptoms of dizziness, weakness, nausea, vomiting, dyspnea, etc. occurred during drug intake and further to analyze the relationship between adverse reactions and drugs by Noah's assessment scale method.
6. Noah's Assessment Scale (18). It consists of 10 medical questions related to adverse drug reaction (ADR) with pre-set scores and is mainly used to evaluate and determine the causal relationship between drug use and ADR. According to the evaluation results of Noah's assessment scale, the causal relationship can be clearly classified into four categories: ① total score ≥ 9, indicating that the causal relationship between the drug and the ADR is certain, i.e., confirmed by objective evidence and quantitative test data; ② total score of 5–8, which is probably related, i.e., supported by objective evidence or quantitative test results; ③ total score of 1–4, which is possibly related, i.e., a situation that can neither be fully confirmed nor completely denied; ④ total score of ≤ 0, suspicious, i.e., a situation that is incidental or basically unrelated.

### Statistical methods

SPSS 27.0 was used for statistical analysis. All statistical tests were performed using a two-sided test, with a *P*-value of <0.05 indicating that the differences were statistically significant. The measurement data, such as age, disease duration, and various scores, were tested for normality using P-P plots and K-S tests, those meeting the normal distribution were expressed as mean ± standard deviation, and those not meeting the normal distribution were described by median and quartile M (P25, P75). Count data, such as gender and co-morbidity, and grade data, such as MGFA level and PSQI score components 2, 5, and 7, were expressed as number of cases (percentage). The difference between PSQI score, MG-ADL score, GAD-7 score, PHQ-9 score, and baseline was statistically described for the enrolled population at the end of the 4th week of treatment. The sample did not satisfy normal distribution, and the paired Wilcoxon rank-sum test was used to analyze whether the differences in PSQI scores, MG-ADL scores, GAD-7 scores, and PHQ-9 scores were statistically significant relative to baseline after treatment. The Wilcoxon rank-sum test was applied to the scores of PSQI components 2, 5, and 7 (grade information) at the baseline period and at the end of the 4th week of treatment. ADRs were assessed for the occurrence of adverse drug events during treatment using Noah's assessment scale, and the ratio of the number of patients with ADRs to the number of patients available for safety evaluation was used to express the incidence of adverse events.

## Results

1. Seventy-five patients met the inclusion criteria. Among them, 45 patients were administered zolpidem, and 30 patients were administered zopiclone.
2. The baseline data, sleep status, and myasthenia gravis status at the first visit and baseline and treatment of eligible patients (Table 1).
3. Changes in the scores (PSQI, MG-ADL, GAD-7, and PHQ-9) at the end of the 4th week of treatment compared with baseline.

**Table 1:** The baseline characteristics of patients.

	**Group**	**Patients (*n* = 75)**
Gender	Male	33 (44.0%)
	Female	42 (56.0%)
Age (years)		56.6 (34.3, 66.4)
Disease course (months)		29.8 (6.5, 50.0)
MGFA at first visit	I	49 (65.3%)
	IIa	9 (12.0%)
	IIb	12 (16.0%)
	IIIa	4 (5.3%)
	IIIb	1 (1.3%)
	IV, V	0 (0%)
MGFA at baseline	I	72 (96.0%)
	IIa	2 (2.7%)
	IIb	1 (1.3%)
	IIIa/b, IV, V	0 (0%)
Co-morbidity	Diabetes	3 (4.0%)
	Hypertension	8 (10.7 %)
	Cardiovascular disease	2 (2.7%)
	History of thymus disease	19 (25.3%)
	Thymus surgery	16 (21.3%)
Prednisone (mg)	Total load (Total disease course)	13,638 (2,520, 20,310)
	Average daily load (Total disease course)	30 (16, 45)
	Average daily load (within observation)	31 (10, 50)
Pyridostigmine bromide (mg)	Total load (Total disease course)	41,220 (19,980, 168,300)
	Average daily load (Total disease course)	180 (180, 199)
	Average daily load (within observation)	180 (60, 180)
MG-ADL		0 (0, 1)
PSQI		15 (13, 15)
GAD7		4 (3, 5)
PHQ9		6 (5, 8)
PSQI component 2	0	0 (0.0%)
	1	1 (1.3%)
	2	31 (41.3%)
	3	43 (57.3%)
PSQI component 5	0	0 (0.0%)
	1	55 (73.3%)
	2	20 (26.7%)
	3	0 (0.0%)
PSQI component 7	0	0 (0.0%)
	1	11 (14.7%)
	2	32 (42.7%)
	3	32 (42.7%)

As seen in Table 2-1, the PSQI score, MG-ADL score, GAD-7 score, and PHQ-9 score decreased significantly at the end of treatment compared with baseline, with a statistically significant difference of a *P*-value of <0.05.

**Table 2-1:** Scores at the end of follow up period of treatment compared with baseline.

	**Baseline**	**End of follow up**	**Change of scores**	***Z-*value**	***P-*value**
PSQI score	15 (13, 15)	9 (7, 12)	5 (3, 7)	−7.439	<0.001
PSQI score range	11–17	4–15	0–11		
MG-ADL score	0 (0, 1)	0 (0, 0)	0 (0, 1)	−3.317	<0.001
MG-ADL score range	0–1	0–1	0–1		
GAD-7 score	4 (3, 5)	4 (2, 5)	0 (0, 1)	−4.366	<0.001
GAD-7 score range	0–9	0–8	−2–3		
PHQ-9 score	6 (5, 8)	3 (2, 5)	3 (2, 4)	−7.500	0.00
PHQ-9 score range	3–9	0–8	0–5		

As seen in Table 2-2, PSQI component 2 (time to sleep), component 5 (sleep disturbance), and component 7 (daytime function) scores decreased at the end of treatment compared with baseline, with a statistically significant difference of a *P*-value of <0.001.

**Table 2-2:** PSQI component 2, 5, 7 scores at the end of follow up period of treatment compared with baseline.

**Time**			**Baseline**	**End of follow up**		**Baseline**	**End of follow up**		**Baseline**	**End of follow up**
**Number of cases**		**75**								
PSQI score	0	Component 2: Time to sleep	0 (0%)	12 (16.0%)	Component 5: Accumulating problems	0 (0%)	4 (5.3%)	Component 7: Daytime function	0 (0%)	21 (28.0%)
	1		1 (1.3%)	34 (45.3%)		55 (73.3%)	71 (94.7%)		11 (14.7%)	48 (64.0%)
	2		31 (41.3%)	23 (30.7%)		20 (26.7%)	0 (0%)		32 (42.7%)	6 (8.0%)
	3		43 (57.3%)	6 (8.0%)		0 (0%)	0 (0%)		32 (42.7%)	0 (0%)
*Z-*value			−7.241			−4.899			−7.224	
*P-*value			<0.001			<0.001			<0.001	

### Safety analysis

The degree of adverse events was mild in all cases (Table 3). There was no case withdrawal from the study due to adverse events. There was no serious adverse event. All patients had no significant infections during the study period, no dose adjustment of prednisone or pyridostigmine bromide was applied during the follow-up period, and no respiratory distress happened.

**Table 3:** Occurrence of adverse events after treatment and their relationship.

**Total number of cases**	**Adverse reactions**		**Noah's assessment scale method**	
	**Number of cases**	**Symptoms**	**Score**	**Evaluation**
75	6 (8%)	Dizziness	5	Probable
	3 (4%)	Drowsiness	5	Probable
	2 (2.7%)	Weak	3	Possible
	1 (1.3%)	Nausea	5	Probable

## Discussion

In this study, a fixed dose of non-benzodiazepine (zolpidem 10 mg/zopiclone 7.5 mg per night) was applied to treat patients with stable MG combined with insomnia. MG symptoms were stable at baseline. In total, 63 of 75 patients stayed stable (MG-ADL ≤ 1) for more than 1 month before treatment. The rest of the patients (12/75) were ocular MG with stable eye symptoms. Compared with baseline, PSQI and MG-ADL scores were significantly reduced at the end of the 4th week of treatment (*P* < 0.05), and none of the cases had elevated MG-ADL scores or dyspnea, indicating that the treatment was safe and effective. The goal of insomnia treatment is to improve patients' nocturnal and daytime symptoms. The main evaluation indicators include sleep latency, wake frequency, awakening after falling asleep, total sleep time, as well as the frequency and nature of complaints, distress, and other daytime symptoms (19). The PSQI component 2, 5, and 7 scores at the end of the 4th week of treatment were also significantly reduced from the baseline (*p* < 0.05), indicating that non-benzodiazepine sleep-promoting treatment not only improved sleep latency and maintenance but also brought daytime benefits. At the end of the 4th week of treatment, GAD-7 and PHQ-9 scores were significantly reduced from baseline (*P* < 0.05), indicating that non-benzodiazepine hypnotics did not increase the risk of anxiety and depression, and it is possible that improved sleep resulted in improved quality of life. These in turn improved anxiety and depression symptoms.

A previous study found that glucocorticoid use was an associated factor for MG combined with insomnia. High-dose (>15–30 mg/d) prednisone was more likely to cause insomnia than low doses (≤7.5 mg/d), while pyridostigmine bromide dosage was not significantly associated with affective disorders (2). In our study, the average daily load of prednisone is high dose. This proved the effectiveness of applying non-benzodiazepine hypnotic drugs in patients with higher prednisone dosage. The results of this study could be expanded for treatment for more active or severe MG patients.

There are some limitations in this study. Further stratified analyses can be performed, such as different types and doses of non-benzodiazepines, different ages, MG patients with different MG-ADL scores, different prednisone load, and different insomnia type (chronic/acute/other) stratified analyses. Biomarkers for insomnia and objective sleep measurement could be included for detailed analysis. Through self-control before and after treatment, and evaluation of several scales such as PSQI and MG-ADL, this study tentatively confirmed that the continuous application of non-benzodiazepine hypnotics (zolpidem 10 mg qn or zopiclone 7.5 mg qn) for 4–5 weeks in the treatment of patients with stable MG (MG-ADL score ≤ 1) combined with insomnia could shorten sleep latency, prolong sleep duration, improve sleep quality, and improve daytime function in a safe and effective manner.

Psychiatric co-morbidity is common in patients with myasthenia gravis. It is unclear whether MG combined with insomnia is primary insomnia or co-morbid insomnia, and the exact relationship between MG and psychiatric co-morbidity is an area for further research (4). The relationship between MG and psychiatric co-morbidity needs further research. Hypnotic medications should only be used intermittently and short-term until non-pharmacologic treatments help change the patient's sleep routine, and first-line treatment of insomnia should always include psychotherapy (including sleep hygiene education, stimulus control therapy, sleep restriction therapy, cognitive therapy, and relaxation therapy). For patients who need pharmacologic treatment, first-line medications include non-benzodiazepine hypnotics, depending on patients' needs and co-morbidities (20).

## Data availability statement

The raw data supporting the conclusions of this article will be made available by the authors, without undue reservation.

## Ethics statement

The studies involving humans were approved by Ethics Committee of Peking Union Medical College Hospital (Ethical Approval Number: I-22PJ189). The studies were conducted in accordance with the local legislation and institutional requirements. The participants provided their written informed consent to participate in this study.

## Author contributions

JY: Data curation, Formal analysis, Methodology, Project administration, Resources, Validation, Writing—original draft, Writing—review and editing. MY: Data curation, Formal analysis, Investigation, Methodology, Project administration, Software, Writing—original draft, Writing—review and editing. ML: Data curation, Methodology, Resources, Writing—original draft. YH: Data curation, Methodology, Resources, Writing—original draft. JS: Data curation, Formal analysis, Investigation, Methodology, Project administration, Writing—original draft. YT: Data curation, Funding acquisition, Methodology, Resources, Writing—review and editing. QF: Data curation, Resources, Writing—original draft. ZZ: Data curation, Resources, Writing—original draft. YG: Conceptualization, Formal analysis, Funding acquisition, Investigation, Methodology, Supervision, Validation, Visualization, Writing—review and editing. LC: Funding acquisition, Resources, Supervision, Writing—review and editing.
